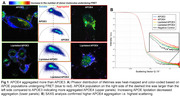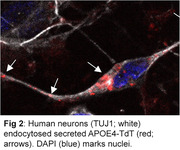# Studying APOE Aggregation in Live Cells and Human Neurons

**DOI:** 10.1002/alz.092614

**Published:** 2025-01-03

**Authors:** Bilal Ersen Kerman, Jason A Junge, Marina Fayzullina, Jinliang Wang, Lucia Z Yang, Cristiana Joy Meuret, Jerome Mertens, Paul Seidler, Scott Fraser, Hussein N Yassine

**Affiliations:** ^1^ USC Keck School of Medicine, Los Angeles, CA USA; ^2^ University of Southern California, Los Angeles, CA USA; ^3^ UC San Diego School of Medicine, La Jolla, CA USA; ^4^ University of Washington‐Seattle, Seattle, WA USA; ^5^ USC Mann School of Pharmacy and Pharmaceutical Sciences, Los Angeles, CA USA

## Abstract

**Background:**

Human Apolipoprotein (APOE) has three isoforms, *ε2*, *ε3*, and *ε4* among which *ε4* (*APOE4*) confers the highest risk for late‐onset Alzheimer’s disease (AD). APOE4 is also the most prone to aggregate among APOE isoforms. Current evidence strongly suggests that APOE aggregation leads to neuronal dysfunction and eventually to AD. APOE4 increases amyloid plaques and neurofibrillary tangles and decreases synapses and neuronal survival. These phenotypes are alleviated by decreasing APOE4 aggregation.

**Method:**

We analyzed APOE aggregation using fluorescence lifetime imaging microscopy (FLIM) in combination with Forster resonance energy transfer (FRET). APOE isoforms tagged with E2GFP or mRuby2 were within 10 nm or less, i.e. in an aggregate the fluorescence lifetime of the donor was shortened and signal intensity was diminished which was monitored using FLIM‐FRET and analyzed using the Phasor approach. APOE aggregation was also confirmed by using small‐angle X‐ray scattering (SAXS) of the Sarkosyl extracts of the cells. Secreted APOE‐Tdtomato was isolated from BHK cell media and analyzed by ion mobility assay. Human neurons were incubated with APOE‐Tdtomato isolated from the media.

**Results:**

APOE4 aggregated more than APOE3 in living BHK cells as shown via FLIM‐FRET microscopy. APOE4 aggregated less when its lipidation was induced by ABCA1 expression in line with data published by us and others. These results were confirmed via SAXS. APOE was secreted in HDL particles from BHK cells both in the presence or absence of ABCA1 while ABCA1 increased secretion for both APOE3 and APOE4. Finally, tagged‐APOE secreted to the media was endocytosed by human neurons.

**Conclusions:**

FLIM‐FRET is a feasible method to analyze APOE aggregation in living cells. Isolated APOE can be used to study APOE endocytosis in human neurons.